# Intratubular, Intracellular, and Mitochondrial Angiotensin II/AT_1_ (AT1a) Receptor/NHE3 Signaling Plays a Critical Role in Angiotensin II-Induced Hypertension and Kidney Injury

**DOI:** 10.3389/fphys.2021.702797

**Published:** 2021-08-02

**Authors:** Xiao Chun Li, Chih-Hong Wang, Ana Paula Oliveira Leite, Jia Long Zhuo

**Affiliations:** Tulane Hypertension and Renal Center of Excellence, Department of Physiology, Tulane University School of Medicine,New Orleans, LA, United States

**Keywords:** angiotensin II, AT_1_ receptors/AT_2_ receptors, hypertension, kidney, proximal tubule

## Abstract

Hypertension is well recognized to be the most important risk factor for cardiovascular diseases, stroke, and end-stage kidney failure. A quarter of the world’s adult populations and 46% of the US adults develop hypertension and currently require antihypertensive treatments. Only 50% of hypertensive patients are responsive to current antihypertensive drugs, whereas remaining patients may continue to develop cardiovascular, stroke, and kidney diseases. The mechanisms underlying the poorly controlled hypertension remain incompletely understood. Recently, we have focused our efforts to uncover additional renal mechanisms, pathways, and therapeutic targets of poorly controlled hypertension and target organ injury using novel animal models or innovative experimental approaches. Specifically, we studied and elucidated the important roles of intratubular, intracellular, and mitochondrial angiotensin II (Ang II) system in the development of Ang II-dependent hypertension. The objectives of this invited article are to review and discuss our recent findings that (a) circulating and intratubular Ang II is taken up by the proximal tubules *via* the (AT_1_) AT_1a_ receptor-dependent mechanism, (b) intracellular administration of Ang II in proximal tubule cells or adenovirus-mediated overexpression of an intracellular Ang II fusion protein selectively in the mitochonria of the proximal tubules induces blood pressure responses, and (c) genetic deletion of AT_1_ (AT_1a_) receptors or the Na^+^/H^+^ exchanger 3 selectively in the proximal tubules decreases basal blood pressure and attenuates Ang II-induced hypertension. These studies provide a new perspective into the important roles of the intratubular, intracellular, and mitochondrial angiotensin II/AT_1_ (AT_1a_) receptor signaling in Ang II-dependent hypertensive kidney diseases.

## Introduction

Hypertension is a well-established risk factor for morbidity and mortality associated with coronary artery disease, stroke, and chronic kidney disease (Muntner et al., 2018; Whelton et al., 2018; Carey et al., 2019). According to the most recent American College of Cardiology/American Heart Association Guidelines, 46% of the United States’ adults have hypertension and will require antihypertensive treatments in their life time (Calhoun et al., 2008; Lloyd-Jones et al., 2010; Sarafidis et al., 2013; Whelton et al., 2018). Yet, only about 50% of hypertensive patients have their blood pressure adequately controlled with current antihypertensive drugs (Carey et al., 2018, 2019; Muntner et al., 2018; Whelton et al., 2018). Although distal nephron-targeting diuretics, the blockers of the renin-angiotensin-aldosterone system, calcium channel blockers, adrenergic *β* receptor antagonists, and renal nerve radiofrequency ablation are widely used to treat hypertension and prevent target organ damage, some hypertensive patients continue to develop cardiovascular, stroke, and renal injury (Jorde et al., 2000; Calhoun et al., 2008, 2019; Lloyd-Jones et al., 2010; Sarafidis et al., 2013). The mechanisms underlying poorly controlled hypertension and kidney injury and the reasons why it is so difficult to treat these patients still remain incompletely understood. Although circulating Ang II levels are not always elevated in most hypertensive patients, renin inhibitors (O’Brien et al., 2007; Oparil et al., 2007; Lambers Heerspink et al., 2009), ACE inhibitors (Wing et al., 2003; Lloyd-Jones et al., 2010; Muntner et al., 2018; Whelton et al., 2018; Carey et al., 2019), and AT_1_ receptor blockers (ARBs) significantly lower the blood pressure in a large number of hypertensive patients (Casas et al., 2005; Lloyd-Jones et al., 2010; Muntner et al., 2018; Whelton et al., 2018; Carey et al., 2019). However, clinical trials have shown that not all the RAS-targeting drugs or other classes of antihypertensive drugs afford the same degree of antihypertensive effects and cardiovascular and renal protection (Casas et al., 2005; Bomback and Toto, 2009; Lloyd-Jones et al., 2010; Muntner et al., 2018; Whelton et al., 2018; Carey et al., 2019). It is therefore imperative to continue to uncover new mechanisms and targets of hypertension and design new antihypertensive drugs to prevent and treat poorly controlled hypertension and target organ injury.

In this invited article, we review and discuss the evidence and recently published studies supporting our hypothesis that intratubular Ang II and AT_1_ (AT_1a_) receptors in the proximal tubules of the kidney are required for maintaining basal blood pressure homeostasis and for the development of Ang II-induced hypertension and renal injury, and that deletion of AT_1a_ receptors selectively in the proximal tubules will attenuate Ang II-dependent hypertension and renal injury. As the proof of concept studies, we recently used highly innovative proximal tubule-specific, genetically modified mouse models with loss of function (knockout) or gain of function (overexpression) to test this hypothesis and determine: (a) whether intratubular Ang II and AT_1a_ receptors or the downstream target the Na^+^/H^+^ exchanger 3 (NHE3) in the proximal tubules are required for maintaining basal blood pressure homeostasis by regulating the pressure natriuresis response (Li et al., 2018, 2019a, 2020, 2021), (b) whether Ang II and AT_1a_ receptors in the proximal tubules are required for the development of Ang II-infused hypertension by resetting the pressure natriuresis response (Li et al., 2021), and (c) whether deletion of Ang II and AT_1a_ receptors or NHE3 selectively in the proximal tubules attenuates Ang II-dependent hypertension and renal injury (Li et al., 2018, 2019a, 2021). The results obtained from these recent *in vitro* and *in vivo* studies likely provide new insights and perspectives into the potential roles of the intratubular, intracellular, and mitochondrial Ang II/AT_1_ (AT_1a_) receptor signaling in Ang II-dependent hypertensive and kidney diseases. It is hoped that the new knowledge may help stimulate further debates or new studies, and potentially lead to a paradigm shift in our understanding of what roles the proximal tubules and the intratubular Ang II system may play in the pathogenesis of hypertension and renal injury. This new knowledge in turn may help develop novel proximal tubule-targeting drugs to prevent and treat poorly controlled hypertension and kidney injury in humans.

## Intratubular, Intracellular, and Mitochondrial Ang II as a New Paradigm of the Renin-Angiotensin System

**Figure 1:**
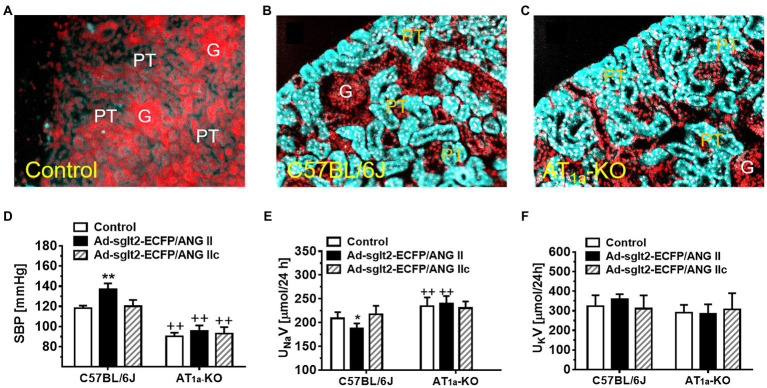
Overexpression of intracellular ECFP/ANG II selectively in the proximal tubule of the kidney in C57BL/6J or AT_1a_-KO mice and its effects on systolic blood pressure and 24 h urinary sodium (U_Na_V) and potassium excretion (U_K_V). **(A)** A representative control C57BL/6 J kidney showing a low level of autofluorescence in the cortex. **(B)** A representative control C57BL/6 J kidney showing overexpression of ECFP/ANG II selectively in the proximal tubule of the superficial cortex in blue-green color. **(C)** A representative AT_1a_-KO mouse kidney also showing overexpression of ECFP/ANG II selectively in the proximal tubule. Red represents DAPI-stained nuclei in the cortex after conversion from blue color. **(D)** Effect on systolic blood pressure. **(E)** Effect on U_Na_V. **(F)** Effect on U_K_V. _**_p < 0.01 vs. control, whereas ^++^*p* < 0.01 vs. ECFP/ANG II overexpression. N = 6–8 for each group. Reproduced with permission (Zhuo et al., 2016).

The RAS is now not only recognized as a circulating or endocrine system (tissue-to-tissue) but also increasingly viewed as a functional paracrine (cell-to-cell) and intracrine (intracellular and/or nuclear) system in the proximal nephron of the kidney (De Mello and Danser, 2000; Cook et al., 2006; Zhuo et al., 2006a; Kobori et al., 2007; Kumar et al., 2008; Li et al., 2008, 2015b). Ang II is the most powerful peptide of the RAS to induce classical cardiovascular, renal, and hypertensive effects by activating AT_1_ (AT_1a_) receptors (Timmermans et al., 1993; De Gasparo et al., 2000; Touyz and Schiffrin, 2000; Carey and Siragy, 2003; Crowley et al., 2005; Higuchi et al., 2007). *In vitro*, sustained stimulation of the AT_1_ (AT_1a_) receptors leads to its desensitization and loss of vasoconstrictive responses to Ang II (Hein et al., 1997; Zhang et al., 1997; Ferguson, 2001). This phenomenon has led a long-held paradigm for G protein-coupled receptor (GPCR) pharmacology that repeated stimulation of GPCR by agonists will not have long-term pharmacological effects because of its receptor desensitization. However, we and others have shown that infusion of Ang II for weeks continues to induce progressive hypertension and target organ kidney injury (von Thun et al., 1994; Zou et al., 1996; Zhuo et al., 2002; Li et al., 2007; Li and Zhuo, 2008b, 2011, 2013), suggesting that this classical paradigm should be revised to include the intracellular system (Kurtz and Gardner, 1998; De Mello and Danser, 2000; Re, 2000; Kumar et al., 2008). Indeed, Ang II is rapidly internalized with AT_1_ (AT_1a_) receptors in target cells, but not all of internalized Ang II is sorted to the lysosome degradation pathway in proximal tubule cells (van Kats et al., 2001; Li et al., 2006, 2007, 2009, 2014; Li and Zhuo, 2014). Some internalized Ang II bypasses the lysosome degradation pathway and is transported to the mitochondria, endoplasmic reticulum, and nucleus, where it continues to induce signaling and long-term transcriptional responses or long-lasting genomic effects by activating mitochondrial and nuclear AT_1a_ receptors (Kurtz and Gardner, 1998; van Kats et al., 2001; Bivona and Philips, 2003; Cottrell et al., 2009; Murphy et al., 2009). We and others have evidence that intracellular administration of Ang II induces the expression of nuclear factor-κB (Brasier et al., 2000; Ruiz-Ortega et al., 2000; Zhuo et al., 2006b, 2016;Schupp et al., 2007; Li and Zhuo, 2008a), monocyte chemoattractant protein 1 (MCP-1; Zhuo, 2004; Li and Zhuo, 2008a; Takahashi et al., 2008), TNF-α (Takahashi et al., 2008), TGF-β1, and NHE3 (Kagami et al., 1994; Wolf et al., 1999; Weigert et al., 2002), and induces $o_{2}^{-}$ production in the mitochondria and nucleus of the proximal tubule cells (Gwathmey et al., 2010a,b; Li et al., 2020). Furthermore, global or proximal tubule-specific overexpression of an intracellular ANG II fusion protein selectively in the proximal tubules of the kidney, Ad-sglt2-ECFP/Ang II (Figure 1), or in the mitochondria of the proximal tubules, Ad-sglt2-mito-ECFP/Ang II, developed antinatriuretic responses and elevated blood pressure by altering the mitochondrial functions (Li et al., 2011b, 2020, 2021; Li and Zhuo, 2013). Overall, these proof of concept studies strongly support a new paradigm of a functional proximal tubule intratubular, intracellular, and mitochondrial Ang II system in the development of hypertension and renal injury.

## Intratubular AT_1_ (AT_1A_) Receptors in the Proximal Tubules Play an Important Role in the Pressure Natriuresis Response and its Resetting in the Development of Hypertension

The pressure-natriuresis response is a central element of the overall feedback mechanism for long-term control of arterial pressure, in which an increase in arterial pressure will lead to a decrease in Na^+^ reabsorption and a natriuresis response in the kidney and restore blood pressure to normal (Roman, 1986; Cowley and Roman, 1996; Hall et al., 1996; Granger et al., 2002; Li et al., 2018, 2019a, 2021). The pressure natriuresis response is reportedly mediated by: (a) inhibition of proximal tubule Na^+^ transport (Moreno et al., 2001; Dos Santos et al., 2004), (b) increase in renal interstitial hydrostatic pressure (Li and Zhuo, 2013), (c) increase in renal medullary blood flow (Roman, 1986; Williams et al., 2007), (d) increase in 20-HETE production (Moreno et al., 2001; Dos Santos et al., 2004; Williams et al., 2007), (e) increase in AT_2_-mediated cGMP production (Siragy and Carey, 1996; Jin et al., 2001, 2004), (f) increased dopamine-induced signaling (Hussain and Lokhandwala, 1998; Banday and Lokhandwala, 2008; Wang et al., 2009), or (g) increased renal nitric oxide (Majid et al., 1993, 1998). None of these factors, however, adequately explains the pressure natriuresis response in hypertension. We reasoned that the proximal tubules are responsible for reabsorbing ~65–~70% of filtered Na^+^ (Wilcox et al., 1992; Wang et al., 2009; Li and Zhuo, 2013) and Ang II exerts a powerful stimulatory effect on proximal tubule Na^+^ reabsorption (Harris and Navar, 1985; Cogan, 1990; Wang and Chan, 1990; Li and Zhuo, 2013). We hypothesized that intratubular Ang II *via* activating AT_1_ (AT_1a_) receptors in the proximal tubules plays a key role in the regulation of the pressure natriuresis response and it is resetting in Ang II-dependent hypertension. Indeed, an impaired pressure natriuresis response has been reported in SHR (Roman and Cowley, 1985; Roman, 1987) and animal models of L-NAME- (Majid et al., 1993; Granger and Alexander, 2000), 2-Kidney, 1-Clip (Rostand et al., 1982), TGR (mRen-2)27- (Gross et al., 1994; Zhuo et al., 1999), and Ang II-induced hypertension (Mattson et al., 1991; Wang et al., 2000; Zhuo et al., 2002; Li et al., 2011a, 2021). Most, if not all, of these hypertension models involve the activation of intratubular Ang II and AT_1_ (AT_1a_) receptors in the proximal tubules, which stimulates proximal tubule Na^+^ reabsorption and induces Na^+^ retention. Nevertheless, the roles of intratubular AT_1_ (AT_1a_) receptors in the proximal tubules in the regulation of the pressure natriuresis response and hypertension have not been investigated using strictly proximal tubule-specific, genetically modified animal models. Although AT_1_ (AT_1a_) receptors were reportedly deleted from the proximal tubules using the phosphoenolpyruvate carboxykinase (*PEPCK*) promoter-driven *Cre* (Gurley et al., 2011) or the androgen-dependent promoter (*KAP2*)-driven *Cre* approach (Li et al., 2011a), the specificity or selectivity of *PEPCK* and *KAP2* to drive *Cre* expression selectively in the proximal tubules remains uncertain. PEPCK is abundantly expressed in the epithelial cells of liver and the digestive system, whereas KAP2 is also expressed extensively in many other androgen-responsive tissues or tubular segments in the kidney (Ding et al., 1997; Li et al., 2008). Despite the lack of specificity, however, these studies were still able to demonstrate an important and lower basal blood pressure phenotype (Gurley et al., 2011; Li et al., 2011b). It may be reasonably argued that the blood pressure phenotype and its response to Ang II in these mutant mouse models may not be due only to the deletion of AT_1a_ receptors in the proximal tubules, but also likely involve the absence of AT_1a_ receptors in other tissues with the expression of PEPCK and KAP2.

**Figure 2:**
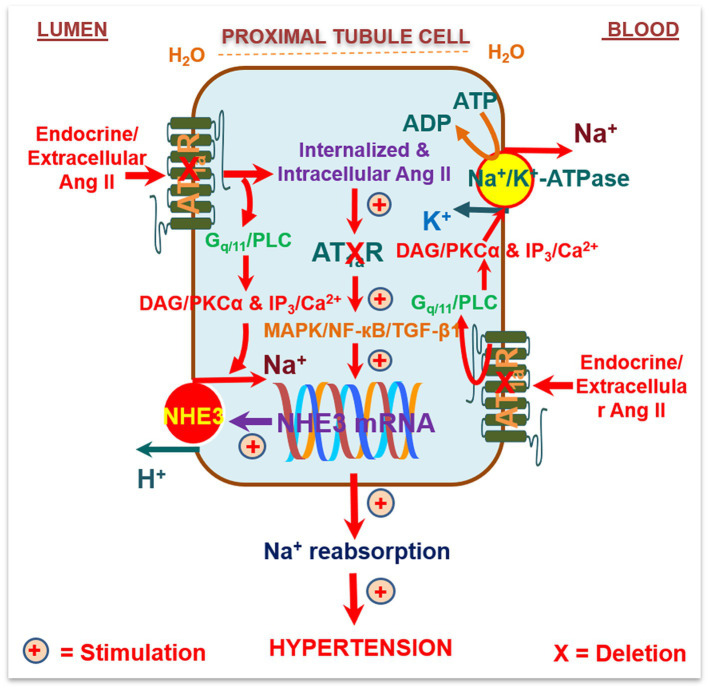
Schematic diagram showing the hypothesis that circulating/extracellular Ang/intratubular II binds to apical (lumen) and basolateral membrane (blood) AT_1a_ receptors, and activates G protein-coupled Gq11/phospholipase C (PLC)/diacylglycerol(DAG)/protein kinase C*α* (PKC*α*) signaling, and/or inositol trisphosphate (IP_3_)/Ca^2+^ signaling pathways. This activated signaling increases the activities and expression of Na^+^/H^+^ exchanger 3 (NHE3), Na^+^/K^+^-ATPase, and other Na^+^ cotransporters in the proximal tubules to increase Na^+^ reabsorption and maintain arterial blood pressure under physiological conditions and elevate it during Ang II-induced hypertension. Alternatively, circulating/extracellular Ang II is taken up by the proximal tubules *via* AT_1a_ receptors and the endocytic receptor protein megalin under physiological conditions and during Ang II-induced hypertension to act as an intracellular/intracrine peptide. The latter will in turn induce long-term transcriptional effects to increase the expression of NHE3, Na^+^/K^+^-ATPase, and other Na^+^ cotransporters in the proximal tubules, promote proximal tubule Na^+^ reabsorption, and elevate blood pressure. As expected, deletion of AT_1a_ receptors selectively in the proximal tubules of the kidney will attenuate circulating/extracellular and intratubular/intracellular Ang II-induced hypertension in PT-*Agtr1a^−/−^* mice. Reproduced with permission (Li et al., 2021).

To overcome the limitation of these technical approaches, we have recently used the *Sglt2-Cre*/*Agtr1a*-foxed recombination to delete AT_1_ (AT_1a_) receptors selectively in the proximal tubules of the kidney and to determine the specific roles of intratubular Ang II and AT_1_ (AT_1a_) receptors in basal blood pressure homeostasis and the development of hypertension induced by circulating or intracellular Ang II (Rubera et al., 2004; Li et al., 2011b, 2021; Rateri et al., 2011). The hypothesis to be tested was that intratubular Ang II and AT_1a_ receptors in the proximal tubules are required for maintaining normal blood pressure and the development of Ang II-induced hypertension. We treated adult male wild-type, global *Agtr1a^−/−^*, and PT-*Agtr1a^−/−^* mice with osmotic minipump infusion of a high pressor dose of Ang II (1.5 mg/kg/day, i.p.), a slow pressor dose of Ang II (0.5 mg/kg/day, i.p.), or with adenovirus-mediated overexpression of an intracellular Ang II fusion protein in the proximal tubules of the kidney for 2 weeks (Figure 2; Li et al., 2021). Deletion of AT_1a_ receptors in the proximal tubules led to a decrease in basal telemetry blood pressure by ~15 ± 3 mmHg in PT-*Agtr1a^−/−^* than wild-type mice, which was ~13 ± 3 mmHg higher than the whole-body *Agtr1a^−/−^* mice. The lower basal blood pressure phenotype was associated with an increase in basal glomerular filtration by ~23.9%, a decrease in fractional proximal tubule Na^+^ reabsorption, and augmented the pressure-natriuresis response and natriuretic responses to salt loading or Ang III infusion in PT-*Agtr1a^−/−^* mice (Li et al., 2021). Furthermore, deletion of AT_1a_ receptors in the proximal tubules attenuated ~50% of Ang II-induced hypertension in PT-*Agtr1a^−/−^* mice, compared with wild-type mice, but completely blocked intracellular Ang II fusion protein-induced hypertension in PT-*Agtr1a^−/−^* mice (Li et al., 2021). Taken together, the results of this study provide new insights into the critical role of intratubular Ang II/AT_1_ (AT_1a_) pathways in the proximal tubules in normal blood pressure control and the development of Ang II-induced hypertension.

However, it remains unknown whether deletion of AT_1a_ receptors selectively in the proximal tubules of the kidney may alter the expression of other peptide receptors, such as AT_1b_, AT_2_, dopamine, or endothelin (ET) receptors alone, or alter other heterodimer receptors in the proximal tubules. Heterodimer Ang II AT_1a_ and D3 dopamine receptors (Zeng et al., 2003) or heterodimer AT_1a_ and endothelin ET_B_ receptors have been reported previously in renal proximal tubule cells of SHR (Zeng et al., 2005). In the absence of AT_1_ (AT_1a_) receptors in the proximal tubules, these receptors may act to inhibit proximal tubule Na^+^ reabsorption, promote the pressure-natriuresis response, and lower the basal blood pressure or help attenuate Ang II-induced hypertension. Further studies are necessary to determine the roles or interactions between intratubular Ang II, dopamine, natriuretic peptide, or ET_B_ receptor signaling pathways in the proximal tubules in blood pressure control and body salt and fluid balance.

## The Proximal Tubule Na^+^/H^+^ Exchanger 3 Plays an Important Role in Intratubular and/or Intracellular Ang II-Induced Hypertension

The NHE3 is well recognized to be the most important Na^+^/H^+^ antiporter member in the proximal tubules of the kidney (Lorenz et al., 1999; Wang et al., 1999; Vallon et al., 2000; McDonough, 2010; Li et al., 2018, 2019a). NHE3 acts directly to extrude H^+^ from proximal tubule cells in exchange for luminal Na^+^ entry, directly contributing to ~25% of active Na^+^ reabsorption, and after generating a luminal Cl^−^ gradient, to drive passive reabsorption of additional >30% of the filtered Na^+^ load in the proximal tubules (Aronson, 1983; Rector, 1983; Schafer et al., 1984; Li and Zhuo, 2013). We and others have shown that global knockout of the *Nhe3* gene in *Nhe3^−/−^* mice decreases Na^+^ reabsorption in the proximal convoluted tubule by 50% and lowers basal blood pressure by about 15 mmHg (Schultheis et al., 1998; Woo et al., 2003; Noonan et al., 2005; Li et al., 2015a,b). Even with the transgenic rescue of the *Nhe3* gene selectively in small intestines of the gastrointestinal tract in tg*Nhe3^−/−^* mice, basal blood pressure remained significantly lower, suggesting that NHE3 in the kidney plays a critical role in maintain basal blood pressure homeostasis (Woo et al., 2003; Noonan et al., 2005; Li et al., 2015b). Indeed, Fenton et al. were instrumental in generating a new kidney-selective *Nhe3^−/−^* mouse model using the *Pax8-Cre/NHE3^loxlox^* approach to determine the role of renal tubule NHE3 in blood pressure regulation (Fenton et al., 2017). This approach appears to be superior to tg*Nhe3^−/−^* mice with the transgenic rescue of the *Nhe3* gene selectively in small intestines (Woo et al., 2003; Noonan et al., 2005; Li et al., 2015b). However, Pax8, paired box gene 8, is still expressed widely in the epithelial cells of the kidney tubules, endocervix, endometrium, ovary, Fallopian tube, seminal vesicle, epididymis, pancreatic islet cells, and lymphoid cells (Poleev et al., 1992; Thompson et al., 2021). Thus, this mouse *Nhe3^−/−^* model may still be considered as a panepithelial cell-specific or whole-kidney tubule-specific *Nhe3^−/−^* model. Despite this limitation, basal blood pressure was found to be 10–20 mmHg lower in *Pax8-Cre/NHE3^loxlox^* mice when fed with low or high Na^+^ diet. Because this basal blood pressure phenotype is largely similar to those of tg*Nhe3^−/−^* mice, the results of this study are consistent with the hypothesis that NHE3 in the kidney plays a critical role in maintaining basal blood pressure homeostasis.

**Figure 3:**
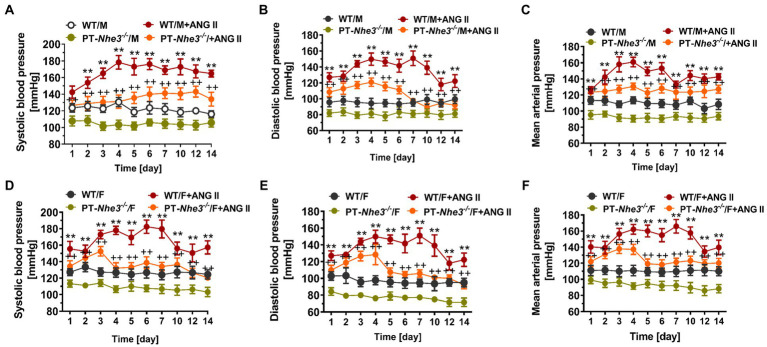
Systolic, diastolic, and mean arterial blood pressure responses to a pressor dose of Ang II infusion, 1.5 mg/kg/day, i.p., *via* osmotic minipump in conscious, adult male (M) and female (F) wild-type (WT) and PT-Nhe3^−/−^ mice, as measured continuously for 14 days using the direct implanted telemetry technique. Please note the time-dependent increases in systolic **(A,D)**, diastolic **(B,E)**, and mean arterial blood pressure responses **(C,F)** in male and female WT mice, and significantly attenuated hypertensive responses to Ang II in male and female PT-Nhe3^−/−^ mice. WT/M, male WT; WT/M+Ang II, male WT with Ang II infusion; PT-Nhe3^−/−^/M, male PT-Nhe3^−/−^; and PT-Nhe3^−/−^/+Ang II, male PT-Nhe3^−/−^ with Ang II infusion. F represents female WT or PT-Nhe3^−/−^ mice. ^**^p < 0.01 vs. WT time-control group and ^++^*p* < 0.01 vs. PT-Nhe3^−/−^ time-control group, respectively. *N* = 5–12 per group. Reproduced with permission (Li et al., 2019a).

Recently, we employed a different and more specific approach to generate a mutant mouse model with proximal tubule-specific deletion of NHE3, PT-*Nhe3^−/−^*, to test our hypothesis on the important roles of NHE3 in the proximal tubules in basal blood pressure control and Ang II-induced hypertension (Li et al., 2018, 2019a). Specifically, PT-*Nhe3^−/−^* mice were generated using the *Sglt2-Cre/Nhe3^loxlox^* approach, whereas Ang II-induced hypertension was induced by Ang II infusion *via* osmotic minipump for 2 weeks (Li et al., 2018, 2019a). We demonstrated that under basal conditions, systolic blood pressure, diastolic blood pressure, and mean arterial blood pressure were significantly lower in male and female PT-*Nhe3^−/−^* than wild-type mice. The lower blood pressure phenotype was again associated with significant inhibition of proximal tubule Na^+^ reabsorption, resulting in significant natriuretic responses and augmented pressure-natriuresis response in PT-*Nhe3^−/−^* mice (Li et al., 2018). As expected, Ang II induced robust hypertension in wild-type mice, but the hypertensive effect of Ang II was attenuated by about 50% in male and female PT-*Nhe3^−/−^* mice (Figure 3). Furthermore, the pressure-natriuresis response was impaired in Ang II-infused wild-type mice but was augmented in male and female PT-*Nhe3^−/−^* mice infused with Ang II (Li et al., 2019a). These results were largely reproduced in wild-type mice infused with Ang II and concurrently treated with an orally absorbable NHE3 inhibitor, AVE-0657 (20 mg/kg/day for 14–28 days), which also significantly attenuated Ang II-induced hypertension in C57BL/6J mice (Li et al., 2019a). Taken together, our studies in PT-*Nhe3^−/−^* mice provide the evidence that NHE3 in the proximal tubules of the kidney plays an important physiological role in proximal tubule Na^+^ reabsorption and basal blood pressure homeostasis, and in the development of Ang II-induced hypertension. NHE3 in the proximal tubules of the kidney may serve as a potential therapeutical target in hypertension associated with the activation of intratubular Ang II system or with increased NHE3 expression in the proximal tubules.

## The Proximal Tubule Ang II and AT_1_ Receptors Play an Important Role in the Development of Hypertensive and/or Renal Ischemia and Reperfusion Injury

In addition to hypertension, renal ischemia and reperfusion (I/R) injury is a leading factor in the pathogenesis of acute kidney diseases commonly developed due to severe hypotension, sepsis, cardiac bypass surgery, and kidney transplantation (Basile et al., 2012; Zuk and Bonventre, 2016; Smith et al., 2019). Renal I/R injury is characterized by temporary loss of blood supply to the kidney, followed by reperfusion, with subsequent activation of intratubular humoral factors, generation of reactive oxygen species (ROS), and initiation of a cascade of proinflammatory and profibrotic responses, and glomerular and tubulointerstitial injury (Basile et al., 2012; Zuk and Bonventre, 2016; Smith et al., 2019). The mechanisms underlying the development of renal I/R injury are extremely complex, involving the RAS (Johnson et al., 1992; Kontogiannis and Burns, 1998; Rodriguez-Romo et al., 2016), ROS (Paller et al., 1984; Morpurgo et al., 1996; Choi et al., 2015), NF-кB (Wan et al., 2011; Xue et al., 2014; Nishikawa et al., 2018), Toll-Like receptor 4 (TLR4; Chen et al., 2011; Trentin-Sonoda et al., 2015; Biancardi et al., 2017), sphingosine-1-phosphate 1 (S1P1) receptor (Bajwa et al., 2010; Deng et al., 2010; Park et al., 2012), and hypoxia-inducible factors (HIF-1α; Tanaka and Nangaku, 2010; Zhu et al., 2011; Luo et al., 2015). However, none of these factors adequately explains how it induces renal I/R injury, and a unified hypothesis may be therefore required. We hypothesize that during the development of renal I/R injury, intratubular and intracellular Ang II and AT_1a_ receptors are activated in the proximal tubules, which play a key role in the pathogenesis of renal I/R by impairing mitochondrial function, and that deletion of AT_1a_ receptors selectively in the proximal tubules attenuates renal I/R injury by blocking AT_1a_-mediated, intracellular Ang II-induced activation of proinflammatory cytokine and chemokine production and profibrotic responses. This hypothesis is supported by studies in which Ang II induced marked vascular, glomerular, and tubulointerstitial macrophage and monocyte infiltration, type IV collagen deposition, and tubulointerstitial fibrosis (Johnson et al., 1992; Kontogiannis and Burns, 1998; Mezzano et al., 2001; Ruiz-Ortega et al., 2002; Rodriguez-Romo et al., 2016). Ang II reportedly activates TLR4 in the proximal tubules of the kidney (Wolf et al., 2006; De Batista et al., 2014; Pushpakumar et al., 2017), S1P1 receptors (Bajwa et al., 2010; Park et al., 2012), and HIF-1α (Tanaka and Nangaku, 2010; Zhu et al., 2011; Luo et al., 2015).

Indeed, intratubular and intracellular Ang II and AT_1a_ receptor signaling pathways in the proximal tubules are expected to be activated by induction of renal ischemia and reperfusion, which may play an important role in inducing mitochondrial dysfunction and kidney injury. Angiotensin II has been linked to mitochondrial dysfunction associated with hypertension and renal injury (de Cavanagh et al., 2007, 2011; Re and Cook, 2010). ARBs improve mitochondrial function and slow the aging process (de Cavanagh et al., 2011), whereas knockout of AT_1a_ receptors prolongs longevity by increasing the number of mitochondria and improving mitochondrial function (Benigni et al., 2009). Whether intratubular Ang II, especially mitochondrial Ang II, *via* activation of mitochondrial AT_1a_ receptors, induces mitochondrial dysfunction in renal I/R injury remains unknown. We hypothesize that intratubular and intracellular Ang II activates cell surface as well as mitochondrial AT_1_ (AT_1a_) receptors to induce activation of the Nox/NADPH or redox-sensitive signaling cascade in the mitochondria (Montezano and Touyz, 2012; Montezano et al., 2015; Li et al., 2020). Increased $o_{2}^{-}$ production by Ang II leads to uncoupling eNOS and activation of proinflammatory, profibrotic, and mitogenic responses, contributing to renal I/R injury (Montezano and Touyz, 2012; Montezano et al., 2015; Li et al., 2020). As one of the Nox/NADPH families, Nox4 is highly expressed in proximal tubule cells (Nlandu et al., 2012; Sedeek et al., 2013). Ang II inhibits the expression of mitochondrial electron transport chain and TCA cycle-modifying genes, induces mitochondrial oxidative stress, and decreases mitochondrial membrane potential (∆ψm) *via* mitochondrial $o_{2}^{-}$ (Kimura et al., 2005; Zhang et al., 2007). Consistent with studies, we and others have localized internalized [^125^I]- and FITC-labeled Ang II and AT_1_ receptors in endosomal, mitochondrial, and nuclear compartments in proximal tubule cells *in vitro* and *in vivo* (Zhuo et al., 2002; Li et al., 2007, 2009, 2020; Gwathmey et al., 2010a,b). Intracellular administration of Ang II to mimic internalized Ang II stimulated intracellular Ca^2+^ mobilization in VSMCs and proximal tubule cells (Haller et al., 1996, 1998; Zhuo et al., 2006a), whereas Ca^2+^ uptake in the mitochondria is closely associated with mitochondrial ATP synthesis (Jouaville et al., 1999; Duchen, 2000) and mitochondrial membrane potential ∆ψm (Hall et al., 2009, 2013). By increasing mitochondrial $o_{2}^{-}$, decreasing NO bioavailability, and impairing mitochondrial function (de Cavanagh et al., 2007; Dikalova et al., 2010), Ang II is expected to contribute to Ang II-induced hypertension and renal I/R injury.

**Figure 4:**
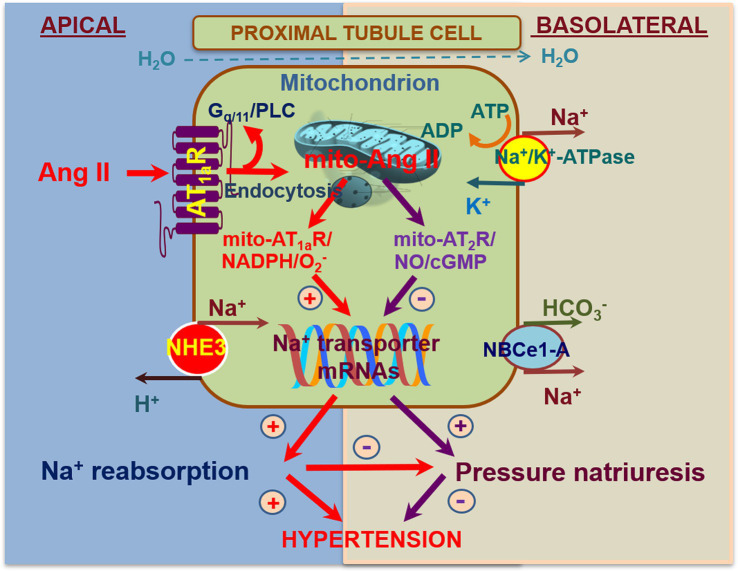
Schematic diagram showing the hypothesis for novel biological and physiological roles of intracellular Ang II system in the mitochondria of the proximal tubules in the regulation of proximal tubule Na^+^ reabsorption and blood pressure homeostasis. In addition to local onsite generation, extracellular (endocrine and paracrine) Ang II is taken up by the proximal tubule cells *via* the AT_1_ (AT_1a_) receptor-mediated mechanism. Some internalized Ang II/AT_1_ receptor complexes bypass the lysosomal degradation pathway and be transported to other intracellular organelles, including the mitochondria and the nucleus, where Ang II activates AT_1_ and/or AT_2_ receptors in the mitochondria to alter mitochondrial oxidative and glycolysis stress responses. This may in turn alter the expression or activity of NHE3 on the apical membranes or Na^+^/K^+^-ATPase on the basolateral membranes in the proximal tubules. Thus, activation of the mito-Ang II/AT_1_/$o_{2}^{-}$ signaling will stimulate proximal tubule Na^+^ reabsorption and elevate blood pressure. Conversely, activation of the mito-Ang II/AT_2_/NO/cGMP signaling by overexpressing AT_2_ receptors selectively in the mitochondria will likely inhibit proximal tubule Na^+^ reabsorption, induce natriuretic response, and lower the blood pressure. Reproduced with permission (Li et al., 2020).

As a proof-of-concept study to demonstrate that the mitochondrial Ang II may directly alter mitochondrial function *via* activation of AT_1_/AT_2_ receptor signaling, we have recently constructed an adenoviral construct encoding a proximal tubule-specific, mitochondria-targeting intracellular Ang II fusion protein, Ad-sglt2-mito-ECFP/Ang II, for its overexpression selectively in the mitochondria of the proximal tubules (Li et al., 2020). We hypothesized that overexpression of Ad-sglt2-mito-ECFP/Ang II selectively in the mitochondria of mouse proximal tubule cells is expected to induce mitochondrial oxidative and glycolytic responses and elevates blood pressure *via* the Ang II/AT_1a_ receptor/$o_{2}^{-}$/NHE3-dependent mechanisms. The expression of mito-ECFP/Ang II in the mitochondria of the proximal tubules was confirmed by the colocalization with MitoTracker Red FM or TMRM in the proximal tubules (Li et al., 2020). *In vitro*, mito-ECFP/Ang II markedly increased oxygen consumption rate (OCR) as an index of mitochondrial oxidative response and extracellular acidification rate (ECAR) as an index of mitochondrial glycolytic response. As the AT_1_ blocker losartan and a mitochondria-targeting superoxide scavenger mito-TEMPO blocked, whereas the nonselective NO inhibitor L-NAME alone increased, the mito-ECFP/Ang II-induced OCR and ECAR responses, our results suggest that mitochondrial Ang II may directly activate AT_1_ receptors to induce $o_{2}^{-}$ production in the mitochondria of proximal tubule cells (Li et al., 2020). In the kidney, overexpression of mito-ECFP/Ang II selectively in the mitochondria of the proximal tubules moderately increased systolic blood pressure by 12 ± 3 mmHg, and the blood pressure-elevating effect of mito-ECFP/Ang II was attenuated in PT-*Agtr1a*^−/−^ and PT-*Nhe3*^−/−^ mice. Interestingly, overexpression of AT_2_ receptors selectively in the mitochondria of the proximal tubules induced moderate natriuretic responses in PT-*Agtr1a*^−/−^ and PT-*Nhe3*^−/−^ mice. Taken together, these results provide new evidence for a physiological role of proximal tubule mitochondrial Ang II/AT_1a_/superoxide/NHE3 and Ang II/AT_2_/NO/NHE3 signaling pathways in maintaining blood pressure homeostasis (Figure 4; Li et al., 2020). Whether intracellular Ang II *via* AT_1_ (AT_1a_) or AT_2_ receptors in the mitochondria induces similar mitochondrial responses in other cells or tissues remains unknown. Given the important roles of Ang II in inducing mitochondrial dysfunction in hypertensive, cardiovascular, and kidney diseases, further studies using innovative, mitochondria-targeting approaches to determine the direct roles of Ang II and underlying mechanisms in the mitochondria are necessary.

## The Mitochondrial Protein Sirtuin 3 Plays an Important Role in the Proximal Tubules in Ang II-Induced Hypertension and Renal I/R Injury

Sirtuin 3 (*SIRT3*) is a member of the sirtuin family of protein deacetylases and plays important roles in maintaining mitochondrial function in humans (Miyazaki et al., 2008; Gao et al., 2014; Kitada et al., 2014). *SIRT3* is primarily localized in the mitochondria matrix, where it acts as a mitochondrial NAD^+^-dependent protein deacetylase to regulate mitochondrial function (Onyango et al., 2002; Kitada et al., 2014; Liu et al., 2015). The primary roles of *SIRT3* in the mitochondria include anti-oxidative, anti-aging, anti-inflammation, and blood pressure-regulating effects by decreasing ROS/O_2_^−^ production *via* activation of long chain fatty acyl-CoA dehydrogenase, succinate dehydrogenase, and NADH dehydrogenase (Onyango et al., 2002; Schwer et al., 2002; Ahn et al., 2008; Someya et al., 2010; Kitada et al., 2014; Liu et al., 2015). By contrast, Ang II acts as an important pro-oxidative, pro-growth, proinflammatory, and hypertensive peptide in part by suppressing *SIRT3* expression in the mitochondria, whereas global deletion of AT_1a_ receptors reportedly increases the expression of *SIRT3* (Benigni et al., 2009). *SIRT3* also appears to be protective against acute kidney injury by improving mitochondrial dynamics (Morigi et al., 2015). However, the roles of mitochondrial *SIRT3* in the proximal tubules in Ang II-induced hypertension and renal injury have not been investigated using mouse model with proximal tubule-specific knockout of *SIRT3*.

We have recently tested the hypothesis that genetic deletion of *SIRT3* selectively in the proximal tubules of the kidney aggravates Ang II-induced hypertension in proximal tubule-specific *SIRT3* knockout mice, PT-*SIRT3^−/−^*. PT-*SIRT3^−/−^* mice were generated using the *SGLT2-Cre/SIRT3-loxP* approach (Li et al., 2019b). Ang II-dependent hypertension was induced by infusing a slow pressor dose of Ang II, 0.5 mg/kg/day, i.p., and a 2% Na^+^ diet for 2 weeks, and compared the hypertensive effect in adult male wild-type and PT-*SIRT3^−/−^* mice. Interestingly, basal systolic, diastolic, and mean arterial pressure were significantly lower, whereas urinary Na^+^ excretion was significantly higher in PT-*SIRT3^−/−^* mice than WT mice, without altering urinary K^+^ excretion (Li et al., 2019b). Furthermore, deletion of *SIRT3* selectively in the proximal tubules of the kidney significantly augmented Ang II-induced hypertension in PT-*SIRT3^−/−^* mice (Li et al., 2019b). Further studies are ongoing to test whether genetic deletion of mitochondrial *SIRT3* in the proximal tubules of the kidney aggravates Ang II-induced hypertension by impairing the pressure-natriuretic response and inducing Na^+^ retention in PT-*SIRT3^−/−^* mice.

## Perspectives

In summary, preclinical animal and human clinical studies over the last few decades have firmly established the important role of the kidney in blood pressure regulation and the development of hypertension by controlling urinary Na^+^ excretion (Cowley and Roman, 1996; Hall et al., 1996; Carey and Siragy, 2003; Crowley et al., 2005; McDonough, 2010; Coffman, 2011; Li and Zhuo, 2013). However, the precise renal mechanisms involved and the relative contributions of renal hemodynamics and tubular transporter systems to basal blood pressure homeostasis and the development of hypertension remain incompletely understood. Indeed, although the loop of Henle- or distal tubule-targeting diuretics has been widely prescribed as a first line of antihypertensive drug in humans, some hypertensive patients still have difficulty in controlling their blood pressure and preventing target organ complications even treated with three different classes of antihypertensive drugs (Casas et al., 2005; Bomback and Toto, 2009; Lloyd-Jones et al., 2010; Muntner et al., 2018; Whelton et al., 2018; Carey et al., 2019). The mechanisms responsible for poorly controlled hypertension remain to be further studied. Based on recent studies from our and other’s laboratories, we hypothesize that the intratubular, intracellular, and mitochondrial Ang II/AT_1a_/NHE3 signaling pathways in the proximal tubules of the kidney may serve as new renal mechanisms and therapeutic targets at least in hypertension and kidney diseases associated with activation of the intratubular renin-angiotensin system. This hypothesis is supported by our recent studies using novel mutant mouse model with proximal tubule-specific deletion (loss of function) or overexpression (gain of function) of major components of the intratubular RAS in the kidney (Li et al., 2018, 2019a, 2020, 2021). Specifically, we have used the state-of-the-art *SGLT2-Cre/LoxP* approach to delete AT_1a_ receptors (Li et al., 2021), the major Na^+^ transporter NHE3 (Li et al., 2018, 2019a), or a key mitochondrial protein *SIRT3* selectively in the S1 and S2 segments of the proximal tubules in the kidney (Li et al., 2019b). Since proximal tubule-specific deletion of AT_1a_, NHE3, or *SIRT3* decreases basal blood pressure, and attenuates or augments Ang II-induced hypertension, we conclude that intratubular Ang II *via* AT_1a_, NHE3, or *SIRT3* in the proximal tubules plays an important role in maintaining basal blood pressure and the development of hypertension and kidney injury. We believe that these studies are highly significant and clinically relevant, and the new knowledge may lead to a paradigm shift on understanding new renal mechanisms of hypertension and kidney injury, and help develop proximal tubule-targeting drugs to treat poorly controlled hypertension and kidney diseases.

## Author Contributions

JZ and XL drafted, reviewed, and finalized the manuscript. All authors contributed to the article and approved the submitted version.

## Conflict of Interest

The authors declare that the research was conducted in the absence of any commercial or financial relationships that could be construed as a potential conflict of interest.

## Publisher’s Note

All claims expressed in this article are solely those of the authors and do not necessarily represent those of their affiliated organizations, or those of the publisher, the editors and the reviewers. Any product that may be evaluated in this article, or claim that may be made by its manufacturer, is not guaranteed or endorsed by the publisher.
